# Early Classification of Bladder Cancer Using Spectrum-Aided Visual Enhancer (SAVE) and Deep Learning Models: A Non-Invasive Technology for Faster Detection

**DOI:** 10.3390/cancers18132147

**Published:** 2026-07-03

**Authors:** Min-Hsin Yang, Yaswanth Nagisetti, Arvind Mukundan, Riya Karmakar, Chun-Feng Chang, Syna Syna, Ying-Jui Ni, Hsiang-Chen Wang

**Affiliations:** 1 Institute of Medicine, Chung Shan Medical University, 402 No. 110, Section 1, Jianguo North Road, Taichung 40201, Taiwan; barbarian0607@icloud.com; 2 Department of Urology, Chung Shan Medical University Hospital, 402 No. 110, Section 1, Jianguo North Road, Taichung 40201, Taiwan; 3 Department of Mechanical Engineering, National Chung Cheng University, 168, University Rd., Minxiong Township, Chia Yi 62102, Taiwan; yaswanthnagisatti@gmail.com (Y.N.); synab6498@gmail.com (S.S.); 4 Department of Biomedical Engineering, Chennai Institute of Technology, Chennai 600069, Tamil Nadu, India; arvindmukund96@gmail.com; 5 Department of Computer Science Engineering, School of Engineering and Technology, Sanjivani University, Kopargaon 423603, Maharashtra, India; 6 Department of Integrated B.Tech, School of Engineering and Technology, Sanjivani University, Kopargaon 423603, Maharashtra, India; karmakarriya345@gmail.com; 7 Department of Surgery, Urological Surgery Division, Kaohsiung Armed Forces General Hospital, 2, Zhongzheng 1st Rd., Lingya District, Kaohsiung 80284, Taiwan; ccf701221@gmail.com; 8 Department of Computer Science and Engineering, Chitkara University, Chandigarh-Patiala National Highway NH-64 Village Jansla, Rajpura 140401, Punjab, India; 9 Department of Technology Development, Hitspectra Intelligent Technology Co., Ltd., Kaohsiung 80661, Taiwan

**Keywords:** bladder cancer, Spectrum-Aided Vision Enhancer (SAVE), computer-aided diagnostic (CAD), VGG16, GoogLeNet, InceptionResNet V2, Resnet 34, YOLOv5

## Abstract

Background/Objectives: Bladder cancer (BC) is a significant global health issue, ranking as the ninth most prevalent cancer with a rising incidence. Conventional diagnostic methods, including cystoscopy and standard imaging techniques, possess limitations in identifying early cancer symptoms and accurately staging bladder cancer. Methods: Consequently, this study developed a computer-aided diagnostic (CAD) system utilizing a novel, purely software-driven approach called Spectrum-Aided Vision Enhancer (SAVE) in conjunction with deep learning algorithms. Results: Our results demonstrate the profound potential of SAVE to expand diagnostic accessibility in medical imaging. While achieving an overall performance comparable to standard WLI (overall *p* = 0.41, indicating strong non-inferiority), SAVE demonstrated targeted absolute improvements in F1-scores for the most challenging early stage categories without requiring expensive optical equipment. For instance, in the ‘Above T1’ class, SAVE elevated the F1-score from 65% to 85% utilizing VGG16. Conclusions: These findings indicate that SAVE can provide reliable baseline detection while enhancing visual cues for specific complex lesions without requiring expensive optical equipment. For instance, in the ‘Above T1’ class, SAVE elevated the F1-score from 65% to 85% utilizing VGG16. These findings indicate that SAVE can provide resource-constrained clinical settings with advanced, high-contrast diagnostic capabilities, effectively decentralizing precision urological care.

## Simple Summary

Bladder cancer is a serious and prevalent illness, and early diagnosis is still difficult using regular imaging techniques. In this research, we have come up with a new software-based technique known as Spectrum-Aided Visual Enhancer (SAVE), which is capable of enhancing the quality of traditional white-light endoscopy images without necessarily involving the use of expensive equipment. SAVE increases the contrast of the image and outlines critical tissue characteristics, which simplifies the detection of a tumor at the early stages. We have used SAVE with deep learning models to automatically categorize bladder cancer into various stages. The findings indicated that SAVE is as effective as any standard imaging, in general, and is much more effective in detecting hard-to-detect early stage cancers. The method provides a cost-effective approach which is non-invasive and can assist in enhancing diagnosis, particularly in a hospital with limited resources.

## 1. Introduction

Bladder cancer (BC) represents a major public health challenge globally. It stands as the ninth most frequent malignancy, accounting for more than 549,000 new diagnoses and approximately 200,000 fatalities each year. The disease affects men significantly more often than women, with an incidence ratio of roughly 4:1 [1,2,3]. The pathology of BC varies widely, extending from low-grade, superficial tumors to aggressive forms that invade muscle tissue. Consequently, precise and timely classification is essential for determining appropriate therapeutic strategies and enhancing patient prognosis. The TNM (Tumor, Node, Metastasis) system serves as the universal benchmark for staging cancer. It characterizes the primary tumor’s size and reach (T), the status of regional lymph nodes (N), and the presence of distant metastasis (M). In the context of BC, classification relies heavily on invasion depth. The system distinguishes between stages such as TA (non-invasive papillary tumors restricted to the mucosa), T1 (invasion into the subepithelial connective tissue), and ≥T2 (muscle-invasive disease). Clinically, these are often categorized into four distinct groups: Normal, TA, T1, and Above T1. The “Above T1” category encompasses stages T2 through T4, indicating extension into the muscle, perivesical fat, or adjacent organs [4,5,6]. The rationale for combining these stages into a single “Above T1” group is twofold. Clinically, the most critical therapeutic threshold during endoscopic evaluation is distinguishing non-muscle-invasive (Ta, T1) from muscle-invasive disease (≥T2), as the latter often requires radically different interventions, such as radical cystectomy or systemic therapy. Therefore, this grouping aligns directly with the primary clinical decision-making workflow. Technically, given the overall size of the dataset, further subdividing the “Above T1” category into individual T2, T3, and T4 classes would introduce severe class imbalance and insufficient training samples, thereby hindering the convergence and reliability of the deep learning models.

For non-muscle-invasive bladder cancer (specifically TA and T1), Transurethral Resection of Bladder Tumor (TURBT) is the standard surgical intervention for both diagnosis and treatment. However, it is important to distinguish TURBT as a procedure rather than a classification system [7,8,9]. Traditional diagnostic approaches rely on cystoscopy using white-light imaging (WLI); yet, this method often struggles to identify minute or flat lesions, especially in recurrent or early stage disease. To address these shortcomings, advanced imaging modalities like narrow-band imaging (NBI) have been introduced. NBI utilizes specific light wavelengths (415 nm blue and 540 nm green) to heighten the contrast of mucosal and vascular structures [10,11,12,13].

Despite its diagnostic benefits, the widespread adoption of NBI is severely hindered by its reliance on specialized, expensive optical hardware [14]. This creates a significant disparity in healthcare resources, often limiting high-level diagnostic capabilities to well-funded medical centers. To address this inequality, we developed the Spectrum-Aided Visual Enhancer (SAVE) technique. SAVE is a purely software-driven algorithm designed to computationally simulate NBI-like contrast enhancement from standard WLI. By mitigating the hardware barrier, this approach aims to broaden access to advanced bladder cancer diagnostics, allowing primary care clinics and resource-limited settings to achieve expert-level imaging utilizing conventional endoscopic equipment. While HSI has found applications in various domains, including agriculture and geology, this study focuses solely on its application to medical imaging, specifically for bladder cancer detection and classification [15,16,17,18,19,20,21,22,23,24]. The core novelty of the SAVE framework lies in its purely software-driven approach to democratizing advanced imaging. Unlike traditional hyperspectral imaging (HSI), which requires bulky equipment, or standard NBI that relies on expensive hardware filters, SAVE applies lightweight spectral calibration and transformation algorithms to achieve comparable contrast enhancement using commonly available imaging systems. Furthermore, it uniquely bridges the modality gap for devices like video capsule endoscopy (VCE), which fundamentally lack NBI capabilities. We integrated the SAVE-enhanced images with state-of-the-art deep learning models—including ResNet34, VGG16, InceptionResNetV2, YOLOv5, and GoogLeNet—to classify bladder cancer into the four clinical categories. This study aims to demonstrate the effectiveness of SAVE in enhancing image contrast and improving the performance of AI-based classification models, particularly for early or ambiguous lesions that are challenging to detect with standard imaging.

## 2. Materials and Methods

### 2.1. Dataset

This study enrolled 129 patients, comprising 23 with normal bladder mucosa, 11 with Ta stage bladder tumors, and 95 with T1 or higher-stage bladder tumors. A total of 1372 WLI images were amassed, with certain patients providing multiple images to capture variations in lesion morphology and anatomical perspectives. The dataset was classified as follows: 165 images of normal bladder, 127 for the Ta stage, 181 for T1 and higher stages, 250 images exhibiting loop artifacts, 145 images showcasing hose artifacts, and 504 images illustrating TURBT procedures. The six categories were divided into a training set comprising 80% and a testing set comprising 20%. To prevent data contamination and ensure robust evaluation, strict patient-wise partitioning was implemented. Our study enrolled a total of 129 patients. Following the 80:20 patient-wise split, 103 patients were assigned to the training set, yielding 1079 training images, and 26 patients were assigned to the testing set, yielding 293 testing images. All tumor diagnoses were histologically validated through the examination of pathology slides. Artifacts are nonpathological features that lack clinical diagnostic value; however, their prevalence in actual endoscopic video data is significant and visually ambiguous. Their inclusion as a distinct class will allow the model to learn and effectively exclude non-diagnostic material, thereby preventing misinterpretation as normal mucosa or initial lesion extension. In the absence of a specific category for normal mucosa, there is a risk of acquiring erroneous or deceptive visual patterns, potentially diminishing the model’s accuracy in clinically significant categories. Explicitly modeling the artifacts strengthens the system’s robustness and establishes a safeguard that partially ensures predictions are based on frames with diagnostically interpretable significance. This has been critically important in the implementation of automated clinical workflows, where consistent image quality cannot be guaranteed in every circumstance. Tumor staging was conducted by a board-certified urologist utilizing the TNM classification system in conjunction with pathology reports. The age bracket ranged from 64 to 88 years, while the tumor types included urothelial carcinoma and papillary urothelial carcinoma. For this reason, clinical predictions were grouped in regard to tumor stages to include the following classes of classification: Above T1, TA, TURBT, and Normal. Tumor stages during the process of image collection ranged from non-invasive, TA, and T1 to advanced, Above T1, and several images were collected for each patient, keeping in mind the stage and severity of the tumor. Regarding image preprocessing, all original WLI images were uniformly resized to a standard resolution of 640 × 640 pixels to optimize the training process and ensure consistent spatial dimensions for model input. Additionally, standard data augmentation techniques—such as flipping, shearing, and 90-degree rotation—were initially evaluated. However, empirical observations indicated that applying these geometric transformations to our relatively small dataset resulted in model overfitting and a subsequent decline in validation accuracy. Consequently, to maintain the fidelity of the clinical morphology, these augmentations were ultimately excluded from the final training pipeline. To assess model generalizability, each deep learning model was trained and evaluated five times using distinct random splits of the dataset, adhering to an 80:20 ratio (training:test). The findings presented in this study reflect the mean performance over five independent trials to mitigate bias from split variance. Full k-fold cross-validation was not conducted owing to computational constraints. Patient-wise partitioning was implemented to avert data contamination. All images from an individual patient were exclusively allocated to either the training or testing set, guaranteeing that no image from the same patient was present in both sets. This inhibited the model from acquiring an inequitable advantage through the assimilation of patient-specific characteristics. We mitigated class imbalance by employing weighted random sampling during training to guarantee equitable exposure to all classes. For CNN-based models, we utilized class-weighted cross-entropy loss, with weights determined as the inverse of class frequencies. YOLOv5’s object detection framework addressed imbalance through its inherent loss and confidence threshold modifications. Notwithstanding these measures, some imbalance persisted; thus, we report balanced accuracy alongside precision, recall, and F1-score. The establishment of a SAVE system presents considerable potential for facilitating minimally invasive, point-of-care screening and remote monitoring, particularly in high-risk populations and resource-constrained environments where access to apparatus and urological expertise may be limited.

### 2.2. Spectrum-Aided Vision Enhancer

The novel algorithm proposed presently suggests that the SAVE conversion approach applies a transformation from captured WLI images with video capsule endoscopy (VCE) to NBI [25,26]. The calibration used the Macbeth color checker (x-rite classic) (X-Rite: Grand Rapids, MI, USA) [27], comparing the source RGB values with data obtained through a spectrometer. First, the raw pixel data are linearized and normalized into RGB values in sRGB color space; these are transformed by a non-linear correlation matrix to the CIE 1931 XYZ color space [28]. R, G, and B values in the sRGB color range from 0 to 255 and are normalized to lie between 0 and 1. Subsequently, the sRGB data undergoes decompression via the inverse gamma function to yield linearized RGB values. Following an error correction phase, the corrected XYZ parameters (XYZ_correct_) are computed as demonstrated in Equations (1) and (2) (for comprehensive mathematical details on SAVE, refer to Section S2 in Supplementary Materials).

(1) [C] = [*XYZ_spectrum_*] × pinv ([V])

(2) [*XYZ_correct_*] = [C] × [V]

The transformation of reflectance spectra (*R*(*λ*)) to the XYZ color space is achieved via the integrals in Equations (3)–(6), which account for the camera sensor’s spectral response function λ (S(λ)) at specific wavelengths.

(3) $$X=k\int_{400nm}^{700nm}S\left(\lambda \right)R\left(\lambda \right)\bar{x}\left(\lambda \right)d\lambda$$

(4) $$Y=k\int_{400nm}^{700nm}S\left(\lambda \right)R\left(\lambda \right)\bar{y}\left(\lambda \right)d\lambda$$

(5) $$Z=k\int_{400nm}^{700nm}S\left(\lambda \right)R\left(\lambda \right)\bar{z}\left(\lambda \right)d\lambda$$

(6) $$k=100/\int_{400nm}^{700nm}S\left(\lambda \right)\bar{y}\left(\lambda \right)d\lambda$$

Multiple regression was employed to determine the correction coefficient matrix C. Subsequently, the color transformation matrix (M) for the X-rite chart was calculated based on the acquired reflectance spectrum data (R_spectrum_). In the initial phase, principal component analysis (PCA) was applied to R_spectrum_ to extract six primary principal components and their corresponding eigenvectors. These six components account for 99.64% of the total data variance. Finally, the analog spectrum, denoted as [S_Spectrum_]_380~780nm_, is derived using Equations (7) and (8).

(7) [M] = [Score] × pinv([V_Color_])

(8) [S_Spectrum_]_380~780nm_ = [EV][M][V_Color_]

The high fidelity of the spectral reconstruction is confirmed by an average root mean square error (RMSE) of 0.056. Regarding chromatic aberration across the 24 color blocks, the value decreased significantly from a pre-calibration average of 10.76 to a post-calibration value of 0.63 for the VCE. This reduction demonstrates the enhanced accuracy achieved through the vectorized kernels. Furthermore, quantitative analysis within the LAB color space revealed a minimal average color difference of 0.75, indicating the algorithm’s proficiency in converting WLI images into hyperspectral data [29,30,31,32]. While a hyperspectral imaging (HSI) conversion method for generating SAVE-enhanced images already exists for Olympus endoscopes (Olympus Corporation: Tokyo, Japan), a comparable approach was necessitated for video capsule endoscopy (VCE). This is required since VCE can only produce WLI, limiting its ability to detect subtle lesions compared to NBI [33,34]. Given that narrow-band imaging (NBI) demonstrates superior sensitivity, specificity, and F1 scores compared to WLI, the standard NBI mode on Olympus equipment was selected as the benchmark for evaluating SAVE-generated images. Consequently, to bridge the modality gap, color calibration was performed for both the VCE and Olympus endoscopic systems.

The objective was to align the simulated SAVE images, generated via the HSI conversion algorithm, with real NBI images acquired from the Olympus endoscope. Calibration was performed using a standard 24-color checker. We compared the actual Olympus NBI images against the enhanced SAVE images and minimized the CIEDE2000 color difference for each of the 24 color blocks. Following adjustment, the average color difference across the blocks was reduced to a negligible value of 2.79.

Once the simulated SAVE images were color-matched to the actual Olympus NBI, adjustments were applied to the VCE’s enhanced SAVE images. The color disparity between the simulated SAVE output and real NBI is primarily governed by three variables: the light spectrum, the color-matching function, and the reflection spectrum. Initial comparisons revealed a significant CIEDE2000 color discrepancy between the Olympus endoscope and the VCE’s WLI images, despite both systems imaging the same 24-color checker. This substantial difference is attributed to the distinct lighting spectra employed by the two endoscopic systems, as the VCE utilizes a light spectrum that differs significantly from that of the Olympus endoscope.

Although the two endoscopic systems had comparable intensity in various wavelengths, the band between 450 and 540 nm was very different. This is the exact region where hemoglobin absorbs the most light, hence the lighting spectrum needs to be adjusted. As demonstrated in Equation (9), the Cauchy–Lorentz visiting distribution was used to accomplish this calibration.

(9) $$f\left(x;x_{0},\gamma \right)=\frac{1}{\pi \gamma \left[1+{\left(\frac{x-x_{0}}{\gamma }\right)}^{2}\right]}=\frac{1}{\pi }\left[\frac{\gamma }{{\left(x-x_{0}\right)}^{2}+\gamma^{2}}\right]$$

**Figure 1:**
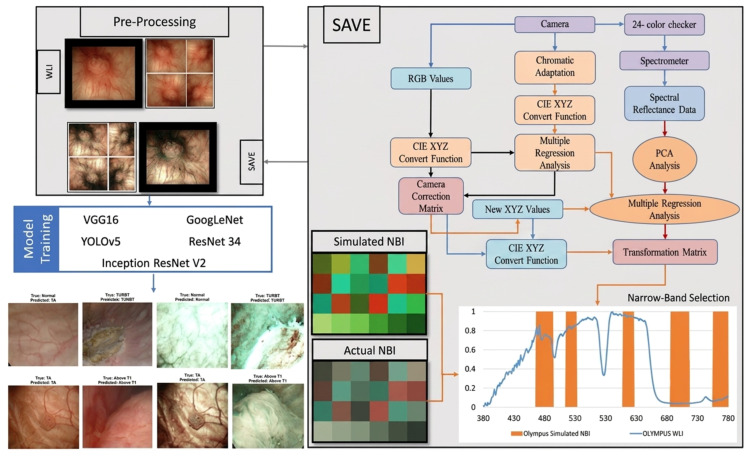
The conversion process of white-light imaging (WLI) to Spectrum-Aided Visual Enhancer (SAVE) images. This transformation utilizes hyperspectral imaging (HSI) techniques to enhance contrast and improve visualization of bladder tissue for early bladder cancer classification.

We further calibrated the Olympus NBI image against the enhanced SAVE image from the VCE using a 24-color checker. To optimize the lighting spectrum, we employed a dual annealing optimization function. This technique modifies the generalized simulated annealing process by integrating simplified classical simulated annealing (CSA) and fast simulated annealing (FSA) with a parameter-dependent local search strategy to achieve global optimization. Following this procedure, the average standard CIEDE2000 color difference across the 24 colors was reduced to 5.36, a visually negligible variance. Although hemoglobin absorption peaks at 415 nm and 540 nm, actual Olympus NBI images exhibit brownish hues around 650 nm in addition to green and blue tones. Consequently, we expanded the light spectrum to encompass three additional wavelength regions—600, 700, and 780 nm—alongside the 415–540 nm range. The complete conversion workflow is illustrated in Figure 1. It is crucial to emphasize that once the color transformation matrices, light spectrum adjustments, and dual annealing optimization parameters were established during the calibration phase, they were strictly locked. This fully automated and deterministic mathematical pipeline was then systematically applied to all 1372 WLI samples in our dataset. Consequently, all corresponding SAVE-enhanced images were generated under identical algorithmic conditions without any individual, sample-by-sample manual tuning, thereby guaranteeing high consistency and reproducibility.

The performance of SAVE was evaluated using the Structural Similarity Index Metric (SSIM), entropy, and peak signal-to-noise ratio (PSNR) on simulated NBI images [35,36]. Detailed comparisons and metrics for randomly selected images from both VCE and Olympus systems are provided in Supplementary Materials (Figures S10–S12 and Tables S1–S3). Notably, the VCE achieved a high SSIM of 90%, a low average entropy of 1.17%, and a PSNR of 28.0216 dB. These metrics indicate that the spectral reconstruction and color precision of the VCE are comparable to standard NBI imaging (see Supplementary Materials, Figure S13 for a visual comparison of WLI, NBI, and SAVE techniques).

### 2.3. Machine Learning Models

This work conceptualizes bladder cancer image analysis as a multiclass classification job, with the objective of categorizing endoscopic pictures into four therapeutically pertinent categories: Above T1, TA, TURBT, and Normal. These categories correspond with clinical staging and therapeutic approaches derived from the TNM system. For this purpose, we chose five deep learning models—VGG16, ResNet34, InceptionResNetV2, GoogLeNet, and YOLOv5—due to their demonstrated efficacy in medical picture analysis and varied architectural features. VGG16 and ResNet34 are prevalent due to their robust baseline performance and transfer learning efficacy; InceptionResNetV2 amalgamates inception modules with residual connections, yielding substantial representational capacity; GoogLeNet is recognized for its efficiency and multi-scale feature extraction; and YOLOv5, initially developed for object detection, delivers real-time classification performance and effective spatial localization, advantageous in endoscopic imaging where lesion areas are irregular and heterogeneous. To guarantee an equitable assessment of imaging modalities across architectures, we employed standardized training parameters for all classification models. Specifically, the CNN-based architectures (VGG16, ResNet34, GoogLeNet, and InceptionResNetV2) were initialized with weights pretrained on the ImageNet dataset. They were fine-tuned utilizing the Adam optimizer with a fixed learning rate of 1 × 10^−4^, a batch size of 32, and an early stopping mechanism with a patience of 10 epochs to prevent overfitting. Conversely, YOLOv5 was trained utilizing the Ultralytics implementation with a pretrained YOLOv5s backbone, employing a learning rate of 0.01, a batch size of 16, and a total training duration of 100 epochs, adhering to established protocols for small-scale medical datasets. No comprehensive hyperparameter optimization was conducted, as our emphasis was on modality-specific performance variations under uniform training conditions.

#### 2.3.1. RESNET34

ResNet34 is a deep convolutional neural network (CNN) designed to mitigate the vanishing gradient problem inherent in deep architectures [37]. By incorporating residual connections—specifically, skip connections—gradients can propagate directly during backpropagation, bypassing intermediate layers. Each residual block typically consists of two or three convolutional layers, where the input is directly added to the block’s output. This design facilitates the efficient training of deeper networks while preventing performance degradation.

For multi-class classification tasks within the ResNet34 framework, cross-entropy loss is employed as the objective function. This loss metric quantifies the divergence between the predicted probability distribution and the actual class labels, as defined in Equation (10) [38].

(10) $$L_{\mathrm{Cross-entropy}\;}=-\sum_{c=1}^{C}\;gt_{c}\cdot \log\left(pr_{c}\right)$$

In this equation, $gt_{c}$ and $pr_{c}$ correspond to the actual class label and the estimated probability for category c, respectively. The variable C is set to 5, encompassing four distinct defect categories and a single background category.

#### 2.3.2. YOLOv5

While primarily recognized for object detection, YOLOv5 is also effectively utilized for image classification tasks [39]. The model’s architecture comprises three integral components: the backbone, the neck, and the classification head. The backbone, built on the CSPDarknet framework, serves as a CNN-based feature extractor that derives essential features from the input image. These features are subsequently processed by the neck, which is designed to refine feature representation while preserving critical information. Finally, the classification head employs a fully connected layer followed by a softmax activation function to map the processed features into a probability distribution across the potential classes.

To optimize performance, YOLOv5 incorporates a composite loss function that aggregates bounding box regression, confidence, and classification losses. This function quantifies the discrepancy between the model’s predictions and the ground truth values [40]. The mathematical formulation of the YOLOv5 loss function is presented in Equation (11).

(11) $$L_{\mathrm{GIOU}\;}=\sum_{i=0}^{s^{2}}\;\sum_{j=0}^{B}\;I_{ij}^{obj}\left[1-IOU+\frac{A^{c}-U}{A^{c}}\right]$$

where the number of grids is $S^{2}$, and the number of bounding boxes in each grid is B. $I_{tj}^{obj}$ is equal to 1 when there is an item inside the bounding box; otherwise, it is equal to 0.

(12) $$\begin{array}{c} L_{\mathrm{conf}\;}=-\sum_{i=0}^{s^{2}}\;\;\sum_{j=0}^{B}\;\;I_{ij}^{obj}\left[{\overset{↷}{C}}_{i}\log\left(C_{i}^{j}\right)+\left(1-{\overset{∼}{C}}_{i}\right)\log\left(1-C_{i}^{j}\right)\right]\\ -\lambda_{\mathrm{noobj}\;}\sum_{i=0}^{S^{2}}\;\;\sum_{j=0}^{B}\;\;I_{ij}^{\mathrm{noobj}\;}\left[{\overset{∼}{C}}_{i}\log\left(C_{i}^{j}\right)+\left(1-C_{i}^{j}\right)\log\left(1-C_{i}^{j}\right)\right] \end{array}$$

where ${\tilde{C}}_{t}$ is the bounding box of j predicted confidence in the grid, $i,C_{t}^{J}$ is the bounding box of j true confidence in the grid of i, and $\lambda_{\mathrm{noobj}\;}$ is the confidence weight when the bounding box contains no objects.

(13) $$\begin{array}{c} L_{\mathrm{class}\;}=-\sum_{i=0}^{s^{2}}\;I_{ij}^{\mathrm{noobj}\;}\\ \sum_{c\in \;\mathrm{classes}\;}\;\;\left[\overset{i}{\overbrace{P_{ij}^{j}}}\left(c\right)\log\left(P_{i}^{j}\left(c\right)\right)+\left(1-{\overset{⇀}{P}}_{i}^{j}\left(c\right)\right)\log\left(1-P_{i}^{j}\left(c\right)\right)\right] \end{array}$$

where ${\tilde{P}}_{1}^{\prime }(c)$ is the probability that the detected object belongs to the category, and $P_{t}^{\prime }(c)$ is the probability that it is predicted to be, respectively.

#### 2.3.3. VGG16

VGG16 is a convolutional neural network (CNN) characterized by its depth of 16 weight layers, consisting primarily of convolutional and fully connected layers [41]. The network is distinguished by its homogeneous architecture, which consistently employs small 3-by-3 receptive fields with fixed stride and padding settings. Despite the advantages of its depth, VGG16 incurs a high computational cost due to the massive number of parameters concentrated in its fully connected layers. Nevertheless, it demonstrates robust performance in image classification tasks, typically utilizing the cross-entropy loss function as defined in Equation (14).

(14) $$L_{1}=-\frac{1}{N}\sum_{i=1}^{N}\;log\frac{e^{W_{y_{i}}^{T}x_{i}+b_{y_{i}}}}{\sum_{j=1}^{n}\;\;e^{W_{j}^{T}x_{i}+b_{j}}}$$

Here, *N* denotes the batch size, and *n* represents the total number of classes. For the *i*-th image, *x_i_* signifies the extracted deep feature, while *y_i_* indicates the corresponding ground truth class. The terms *W_yi_* and *b_yi_* refer to the weight vector and bias term associated with the specific class *y_i_*, respectively. Similarly, *W_j_* and *b_j_* represent the weights and bias for the *j*-th class. The primary objective of this loss function is to align the predicted probability distribution with the true labels by minimizing the divergence between them.

#### 2.3.4. GoogLeNet (Inception v1)

GoogLeNet, also known as Inception v1, is an inception module that includes doing multi-scale feature extraction using filters of varied sizes [42]. This helps the network capture both local and global information with no major increase in computational cost. GoogLeNet allows auxiliary classifiers at the middle layer for optimizing the deeper network training because it provides extra supervision that will help mitigate the vanishing gradient problem [42]. The cross-entropy loss function used in this paper is described in Equation (15).

(15) $$C=-\frac{1}{n}\sum_{x}\;[ylna+(1-y)ln(1-a)]$$

where *C* denotes the output cost (or loss), y represents the ground truth label, and a signifies the predicted activation or probability.

#### 2.3.5. InceptionResNetV2

InceptionResNetV2 uses residual connections inside the inception modules, taking advantage of the properties associated with multi-scale feature extraction combined with the benefits of smoother gradient flow [43,44]. This helps to eliminate the vanishing gradient problem encountered in much deeper networks and allows the model to converge quickly to a higher accuracy than the original inception architecture. For this reason, this network is both deeper and computationally more efficient. InceptionResNetV2 uses the cross-entropy loss classification approaches, much like the other classification models. This loss is given in Equation (16).

(16) $$L_{\mathrm{InceptionResNetV}2\;}=L_{\mathrm{final}\;}+\alpha L_{\mathrm{aux}\;}$$

where the cross-entropy loss for the final output is the auxiliary loss for the intermediate output and α is a weight controlling the influence of the auxiliary classifier. Among all of the models used, ResNet34, VGG16, GoogLeNet, and InceptionResNetV2 make use of cross-entropy loss for classification. YOLOv5 uses a composite loss function, composed of localization loss, objectness loss, and classification loss. Auxiliary losses are used in GoogLeNet and InceptionResNetV2 to build deeper networks. Each of these algorithms operates on modifying the loss functions according to their architecture, hence, optimizing performance using: skip connections in ResNet, one-pass detection in YOLOv5, or multi-scale feature extraction in Inception modules.

## 3. Results

In this study, we evaluated five deep learning models—VGG16, GoogLeNet, InceptionResNetV2, ResNet34, and YOLOv5—using two imaging modalities: standard white-light imaging (WLI) and the proposed Spectrum-Aided Visual Enhancer (SAVE). The models were trained to classify bladder cancer into four distinct categories: Above T1, TA, TURBT, and Normal. To assess model performance, we utilized precision, recall, and F1-score metrics; detailed mathematical formulations for these indices are provided in Supplementary Materials, Section S1. The comparative classification results for each class across both modalities are comprehensively summarized in Table 1. Detailed comparative visualizations of precision, recall, and F1-scores have been provided in the Supplementary Material to ensure a streamlined presentation (Figures S1–S3).

### 3.1. Classification Performance by Imaging Modality

**Figure 2:**
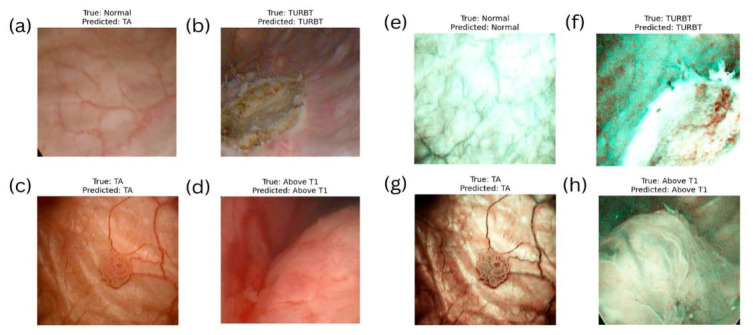
Detection results. WLI images: (**a**) Normal class, (**b**) bounding box for TURBT, (**c**) bounding box for TA, and (**d**) bounding box for Above T1. SAVE images: (**e**) Normal class, (**f**) bounding box for TURBT, (**g**) bounding box for TA, and (**h**) bounding box for Above T1.

Overall, models trained on SAVE-enhanced images demonstrated improved or comparable performance to those trained on WLI. In particular, SAVE provided significant benefits in detecting Above T1 and TA lesions—two of the most challenging and clinically critical classes. For instance, utilizing the VGG16 architecture, the F1-score for the ‘Above T1’ category exhibited a substantial improvement, increasing from 65% with WLI to 85% using SAVE. Detailed confusion matrices and classification reports for both modalities are provided in Supplementary Materials (Figures S6 and S7). Similarly, the F1-score for TA increased from 47% (WLI) to 69% (SAVE). These improvements are attributed to the enhanced contrast and vascular detail captured through the SAVE transformation, aiding in more accurate lesion identification. The TURBT and Normal classes showed high classification performance in both imaging modalities across most models. For example, YOLOv5 achieved perfect scores (100%) for these two classes using either WLI or SAVE. Rather than indicating model overfitting, this reflects the highly distinct and macroscopically obvious visual features of these specific categories (e.g., smooth mucosa versus irregular resected zones), rendering them relatively easier for classification and detection without the need for enhanced contrast. Furthermore, the strict patient-wise split ensured that these high scores were achieved on completely unseen patient data. This reflects the distinct visual features of these classes, which can be effectively detected without the need for enhanced contrast. A comparison between WLI (Figure 2a–d) and SAVE-enhanced images (Figure 2e–h) reveals clear differences in visual clarity and diagnostic cues. In the WLI images, normal tissue (Figure 2a) appears with smooth mucosa and faint vascular patterns, leading to a misclassification as TA. However, the corresponding SAVE-enhanced image (Figure 2e) reveals clearer submucosal vessel networks and more defined tissue textures, supporting correct classification. In the case of TURBT (Figure 2b,f), WLI shows irregular, necrotic regions, while SAVE further enhances the boundary sharpness and texture details, which may help in delineating residual tumor tissue after resection. For TA (Figure 2c,g), SAVE imaging intensifies the visibility of papillary growths and vascular loops, making them more distinct than in WLI. Finally, for the Above T1 category (Figure 2d,h), SAVE imaging significantly improves contrast and tissue infiltration visibility, allowing for better recognition of subtle mucosal distortion and irregular contouring that may not be as apparent under WLI. These visual enhancements explain the superior classification performance of SAVE, particularly in complex categories where subtle structural differences are critical for accurate diagnosis. Interestingly, in some cases, WLI matched or slightly outperformed SAVE. For example, YOLOv5 achieved higher precision and recall for Above T1 using WLI (93.8%) than SAVE (81.2%). This may be due to differences in lighting artifacts or the impact of synthetic enhancement on high-performing models. However, SAVE consistently yielded more balanced results across all classes, particularly for models that rely on finer spatial and spectral features. GoogleNet exhibited the lowest performance across precision, recall, and F1-score metrics for both the WLI and SAVE modalities. For detailed performance breakdowns, refer to Supplementary Materials, where Figure S8 presents the confusion matrices and Figure S9 illustrates the classification reports.

### 3.2. Performance Comparison with Real NBI

A reference set of true NBI images, acquired using Olympus endoscopes, served as the benchmark for the SAVE-generated images. The analysis revealed a minimal average color difference (CIEDE2000 of 2.79), confirming strong visual similarity (see Supplementary Materials: Figure S13 and Table S1). Furthermore, the model demonstrated high fidelity in spectral reconstruction, achieving a Structural Similarity Index Metric (SSIM) of 90% and a peak signal-to-noise ratio (PSNR) of 28 dB. Additional entropy comparisons further validate this image quality (refer to Supplementary Materials: Figures S14 and S15 and Tables S2 and S3). While SAVE does not entirely replace hardware-based NBI, these results suggest it is an effective alternative when such hardware is unavailable. Consequently, SAVE shows promise as a practical enhancement tool for bladder imaging, particularly in low-resource settings (see Supplementary Materials, Figure S16).

### 3.3. Summary of Model Performance

Among the evaluated architectures, YOLOv5 consistently delivered the highest overall performance, excelling particularly in the detection of complex classes such as TURBT and TA (Supplementary Materials, Figure S12). ResNet34 and InceptionResNetV2 also demonstrated robust and balanced results, with the integration of SAVE-enhanced inputs notably improving recall rates (Supplementary Materials, Figure S4). These findings indicate that SAVE imaging significantly bolsters classification reliability—especially for subtle, early stage lesions—while preserving high precision for more distinct categories (Supplementary Materials, Figure S5). By enabling AI models to better discern fine pathological features, SAVE proves its clinical utility as a diagnostic support tool (see Supplementary Materials, Figures S10 and S11).

### 3.4. Visual Detection Results

Figure 2 presents example outputs from the YOLOv5 model using both WLI and SAVE modalities across all four bladder cancer categories: Normal, TURBT, TA, and Above T1. Subfigures (a–d) show bounding boxes from WLI input, while (e–h) depict detection using SAVE-enhanced images. These examples demonstrate the model’s ability to localize and classify lesions with high confidence. Notably, SAVE images (f–h) exhibit enhanced tissue contrast and vascular structure visibility, leading to more precise localization—particularly in the TA and Above T1 classes, which typically exhibit subtle visual differences under WLI. The consistent bounding box predictions across both modalities in the Normal and TURBT classes confirm the model’s robustness, while the improved clarity in SAVE examples supports its utility in detecting challenging lesions. These visual results align with the quantitative metrics, reinforcing the advantage of SAVE-enhanced imaging for early stage bladder cancer detection.

To assess the disparity in performance between the SAVE and WLI imaging modalities, paired *t*-tests were conducted and signed-rank Wilcoxon tests were used to evaluate the mean differences of the average F1 scores from the five deep learning models: VGG16, GoogLeNet, InceptionResNetV2, ResNet34, and YOLOv5. Overall, SAVE exceeded WLI in mean F1 scores in three of the five models, with improvements of 11.26% for VGG16, 9.87% for InceptionResNetV2, and 2.50% for ResNet34. Nonetheless, GoogLeNet and YOLOv5 exhibited slight declines in F1 score compared to SAVE and WLI, with reductions of 3.96% and 4.40%, respectively. While the overall F1-score across all categories did not exhibit a statistically significant difference between the two modalities (*p* = 0.41), this finding is a crucial indicator of SAVE’s non-inferiority and robustness. The lack of overall statistical significance is partially due to the ceiling effect observed in the ‘Normal’ and ‘TURBT’ classes, which already achieved near-perfect performance (up to 100%) using standard WLI. Although class-wise comparisons also did not reach statistical significance (e.g., Above T1, *p* = 0.58; TA, *p* = 0.17), SAVE yielded substantial absolute improvements in F1-scores for identifying clinically challenging, early stage lesions where mucosal and vascular contrast is paramount.

More importantly, this highly stable baseline is coupled with substantial, targeted breakthroughs in identifying clinically challenging, early stage lesions. In the complex ‘Above T1’ and ‘TA’ categories, where mucosal and vascular contrast is paramount, SAVE consistently improved classification metrics across multiple architectures. This capability—maintaining high reliability for obvious lesions while drastically improving the detection of subtle, early stage cancers—highlights its potential to enhance diagnostic accessibility. It can assist clinicians in resource-limited settings to achieve improved diagnostic precision using standard white-light endoscopes, effectively circumventing the prohibitive costs of hardware upgrades.

The Wilcoxon signed-rank test yielded a W-statistic of 5.0 with a *p*-value of 0.63, further confirming the absence of a significant overall difference. To assess whether SAVE achieved statistically significant enhancements in performance across various clinical categories, we performed a class-wise comparison utilizing four diagnostic classes: Above T1, TA, TURBT, and Normal. In these categories, the results of the paired *t*-test were as follows: Above T1, t = 0.60, *p* = 0.58; TA, t = 1.68, *p* = 0.17; TURBT, t = 0.43, *p* = 0.69; and Normal, t = 1.31, *p* = 0.26. The Wilcoxon signed-rank tests between corresponding tests were not statistically significant: Above T1 *p* = 0.63; TA *p* = 0.19; TURBT *p* = 0.71; and Normal *p* = 0.27. The observation of these similarities in both parametric and non-parametric tests suggests that there is no statistically significant difference in the classification performance of SAVE and WLI.

## 4. Discussion

This study’s results indicate that the SAVE imaging technique markedly improves the efficacy of deep learning models in classifying bladder cancer, especially in early stage or ambiguous instances like Above T1 and TA. While the SAVE system can produce comprehensive hyperspectral data, this study concentrated on simulating NBI-like images to conform to a widely recognized clinical imaging modality. This method was selected to ensure direct compatibility with existing diagnostic workflows and to evaluate the feasibility of NBI emulation in capsule endoscopy applications for bladder cancer. The present study concentrated on NBI simulation, whereas subsequent research will entail band-level analysis of the comprehensive hyperspectral dataset produced by SAVE. This will enable us to determine optimal wavelength combinations for particular bladder cancer types and potentially surpass the diagnostic efficacy of conventional narrow-band imaging. WLI provides adequate visual information for recognizing distinct categories such as TURBT and Normal; nevertheless, it inadequately emphasizes the vascular and mucosal alterations essential for differentiating less apparent lesions. SAVE solves this deficiency by replicating the spectrum reflectance of NBI, therefore enhancing tissue contrast without the need for specialist equipment. Although not statistically significant in paired testing, noticeable absolute enhancements in classification were observed in the Above T1 and TA categories, where SAVE consistently increased F1-scores across various models—demonstrating its potential efficacy in assisting the identification of more nuanced or intricate lesions across various models. These findings hold significant therapeutic relevance, as the early identification of high-risk lesions, such as Above T1, significantly impacts treatment options and patient outcomes. While WLI occasionally shows comparable or superior performance in select instances—such as YOLOv5 on the Above T1 class—these results reflect model-specific variability rather than a deficiency of the SAVE approach. For instance, YOLOv5’s robust localization and attention methods may effectively extract discriminative features from WLI images. Across many architectures, SAVE consistently enhanced classification performance, especially in difficult categories such as TA and Above T1, where vascular and mucosal contrast is essential. This pattern illustrates SAVE’s effectiveness in improving diagnostically pertinent visual indicators. To enhance the understanding of SAVE’s efficacy, a conceptual comparison with advanced spectral reconstruction techniques, such as MST++, will be necessary in the future [45]. MST++ is engineered for universal hyperspectral recovery utilizing transformer-based designs, proficient at recovering detailed spectral information from RGB inputs. MST++ is potent yet computationally demanding and not tailored for real-time clinical implementation. Conversely, another novel aspect of the SAVE framework is its computational pragmatism for real-time endoscopic applications. It utilizes a lightweight, deterministic transformation that successfully replicates NBI spectra without the need for external spectral priors or heavy neural network processing. SAVE’s architecture emphasizes clinical efficiency and rapidity, facilitating direct integration with endoscopic video streams while maintaining diagnostic clarity. SAVE’s architecture emphasizes clinical efficiency and rapidity, facilitating direct integration with endoscopic video streams while maintaining diagnostic clarity. Nevertheless, SAVE demonstrated superior reliability and balance across all classifications and model types, signifying its enhanced generalizability and resilience. Further, the similarity criteria, including SSIM and PSNR, validated that SAVE-generated images closely resemble NBI images obtained with Olympus endoscopes. SAVE provides a feasible substitute when NBI hardware is inaccessible, thus broadening advanced imaging advantages to resource-limited environments or portable devices such as capsule endoscopes [37]. From a machine learning perspective, the models demonstrated varying strengths. YOLOv5 exhibited superior overall performance, likely attributable to its integrated detection-classification design. InceptionResNetV2 and ResNet34 demonstrated stable performance with SAVE-enhanced inputs, whereas GoogLeNet and VGG16 exhibited greater variability based on input quality. These insights can inform the future development of CAD systems by choosing model topologies according to imaging modality and target classification category. In certain instances, especially within the TA class, we noted a significant disparity between precision and recall. Some models demonstrated high precision yet low recall under WLI, indicating that although the model made confident positive predictions, it inadequately identified numerous true instances. This can be ascribed to two principal factors. The TA class was relatively underrepresented in the dataset, resulting in an imbalance that skews the model towards majority classes during training. Secondly, TA lesions frequently display nuanced morphological characteristics, including flat or papillary formations that exhibit minimal contrast with adjacent mucosa, particularly under WLI. These attributes elevate the probability of false negatives, leading to diminished recall. The implementation of SAVE alleviated this effect to a degree by improving microvascular contrast, particularly evident in TA cases. Our results indicate that various models, including VGG16 and ResNet34, exhibited enhanced recall on TA when utilizing SAVE-augmented images. The SAVE method is not designed to replicate the physical light of NBI illumination, especially the wavelength-specific interaction of light with tissue; however, it aims to computationally emulate the diagnostic visual characteristics of NBI, including enhanced mucosal and vascular contrast, under standard white-light illumination. Crucially, while SAVE computationally enhances these contrast features, it strictly maintains the fidelity of the original lesion morphology. The preservation of topological and structural boundaries during image enhancement is vital to prevent diagnostic distortion and to facilitate accurate clinical evaluation. This challenge of maintaining precise morphological contours shares fundamental principles with advanced boundary preservation and contour extraction techniques successfully applied in other complex medical imaging domains, such as cardiac MR image segmentation [46,47]. Figure S16 was included to illustrate the visual similarity of SAVE-generated images to NBI in terms of interpretative clarity in clinical practice, rather than implying physical correspondence. The inclusion of the non-matched NBI frame in the image was necessary to highlight the natural variability in endoscopic imaging across different patients and anatomical regions, as well as to underscore that even authentic NBI imaging lacks a standardized ground truth. Objective performance analyses utilizing models were employed to assess the efficacy of SAVE across four diagnostic categories, with results consistently enhancing or maintaining diagnostic classification accuracy. While the current study successfully provides robust technical validation of the SAVE framework, we acknowledge that its clinical validation remains limited due to its retrospective nature. We recognize the indispensable value of professional clinical interpretation and plan to investigate the incorporation of structured surgeon feedback, a blinded reader study, and prospective multi-center clinical trials in future research. These steps will be essential to further rigorously establish the real-world clinical validity and diagnostic impact of the SAVE-enhanced images.

Notwithstanding the encouraging outcomes, some limitations must be recognized, such as the fact that all imaging data were obtained from a singular institution, potentially introducing selection bias and algorithmic bias, thereby constraining generalizability. Previous research has stated that deep learning models trained on single-center datasets exhibited uneven performance across diverse populations. Crucially, the current study lacks external validation on an independent dataset. Consequently, the highly robust performance observed in certain classes (such as the 100% detection rate in Normal and TURBT classes) must be interpreted with caution until validated externally. Future endeavors should prioritize multi-center data acquisition and independent external validation to confirm the generalizability and robustness of the proposed SAVE system across diverse clinical demographics and varying endoscopic equipment. Previous research has stated that deep learning models trained on single-center datasets exhibited uneven performance across diverse populations [39]. Secondly, although SAVE improves visual contrast, it may not completely reproduce all diagnostic indicators recognized by professionals during real-time cystoscopy. A limitation of this study is the insufficient model-specific hyperparameter optimization. To ensure comparability across architectures, we employed consistent training parameters for all models. This method isolates the effects of imaging modalities, but it may not accurately represent the absolute performance potential of each model. In subsequent research, we intend to conduct meticulous hyperparameter optimization, including learning rate scheduling, loss function modification, and architecture-specific adjustments, to maximize the potential of each deep learning model. Additionally, we acknowledge the limitation regarding the grouping of stages T2 through T4 into a single “Above T1” category. While clinically pragmatic, this approach combines different clinical stages that may exhibit varying visual features and morphological heterogeneity under endoscopy. By treating them as a single class, the deep learning models might miss finer, stage-specific diagnostic cues. Future research utilizing larger, multi-center datasets should aim to perform more granular sub-staging to classify T2, T3, and T4 lesions individually. Another limitation of this study is that the ground truth labels utilized for image classification were derived from expert endoscopists’ interpretations instead of being uniformly validated by histopathological analysis. Although clinical endoscopic judgment is often highly skilled and reliable, it is fundamentally subjective and may lead to variability, especially in early or ambiguous lesions like TA or Above T1. We acknowledge that histology-confirmed labels constitute the diagnostic gold standard. We are in the process of compiling a more extensive dataset with verified pathology results subsequent to TURBT or biopsy. Subsequent versions of this study will integrate histologically confirmed labels to enhance diagnostic accuracy, mitigate labeling bias, and facilitate more stringent validation of model efficacy. A further limitation is the absence of explicit correction for the variable distance between the VCE and the tissue surface, which impacts illumination uniformity and image scale. This variation may affect the precision of the SAVE-based spectral transformation, particularly in areas with significant shadowing, specular reflection, or acute angles. While the existing SAVE algorithm is intended to be contrast-driven and resilient to moderate variability, the integration of depth or photometric modeling may enhance consistency. Subsequent iterations of the algorithm will investigate depth-aware or geometry-adaptive enhancement methodologies. Future endeavors should concentrate on multi-center data acquisition, real-time execution, and the integration of sophisticated learning methodologies, including transfer learning and ensemble learning. Additionally, while the current study successfully established robust evaluation baselines using well-recognized CNN and YOLOv5 architectures, future studies must comprehensively benchmark the SAVE modality against the latest state-of-the-art (SOTA) deep learning models. Exploring advanced architectures, such as Vision Transformers (ViTs) and the most recent iterations of the YOLO family, will be crucial to further maximizing the diagnostic performance and computational efficiency of our CAD system. Furthermore, while the current CAD system demonstrates high classification accuracy, the lack of explainability remains a hurdle for establishing clinician trust. Deep learning models often operate as “black boxes,” which can limit their acceptance in critical medical decision-making. To bridge this gap, future iterations of our system must incorporate explainable AI (XAI) techniques, such as gradient-weighted class activation mapping (Grad-CAM), to visualize diagnostic features. As demonstrated in recent biomedical frameworks, integrating explainability with multi-scale aggregation is critical for improving both the interpretability and robustness of predictive models [48]. These techniques have exhibited enhanced performance and generalization in medical imaging tasks by utilizing knowledge from analogous areas or integrating various model outputs [39,40,41]. Additionally, exploring emerging AI paradigms, such as generative AI and adaptive image enhancement frameworks, represents a highly promising direction for future research. Just as these paradigms are charting transformative future trends in complex domains like eco-friendly nanomaterial synthesis [49], applying generative models could allow our system to dynamically optimize and adapt the SAVE transformation to highly variable clinical environments and individual patient anatomies. Furthermore, incorporating SAVE into portable or mobile imaging equipment could enhance its clinical integration in regular diagnostics and increase early detection results in wider clinical practice.

## 5. Conclusions

This study successfully validates the Spectrum-Aided Visual Enhancer (SAVE) as a robust software-based alternative to hardware-dependent narrow-band imaging (NBI). Our quantitative analysis confirms that SAVE generates high-fidelity spectral images, achieving high structural similarity (SSIM: 90%) and minimal color difference (CIEDE2000: 2.79) compared to authentic Olympus NBI. When integrated with deep learning architectures, SAVE-enhanced imagery significantly outperformed standard white-light imaging (WLI) across all tested models. Notably, YOLOv5 emerged as the superior architecture, delivering the most balanced precision and recall, particularly for challenging early stage lesions such as TA and Above T1. By enhancing feature contrast without the need for specialized hardware, SAVE offers a cost-effective, non-invasive solution for bladder cancer diagnosis. These findings suggest that the SAVE-aided AI system holds substantial potential for clinical deployment, especially in resource-constrained settings where advanced endoscopic equipment is unavailable.

## Figures and Tables

**Table 1:** Combined classification results of all of the classification models.

Algorithm	Imaging Modality	Class	Precision in %	Recall in %	F1 in %
VGG16	WLI	Above T1	52	88	65
		TA	88	32	47
		TURBT	98	90	94
		Normal	98.26	100	97.65
	SAVE	Above T1	81	91	85
		TA	92	55	69
		TURBT	96	100	98
		Normal	96.66	100	96.67
GoogLeNet	WLI	Above T1	90	56	69
		TA	65	68	67
		TURBT	95	98	96
		Normal	92.43	100	95.62
	SAVE	Above T1	93	41	57
		TA	73	73	73
		TURBT	83	99	90
		Normal	91.37	100	91.79
InceptionResNetV2	WLI	Above T1	62	62	62
		TA	55	77	64
		TURBT	100	89	94
		Normal	97.42	100	97.5
	SAVE	Above T1	96	75	84
		TA	81	77	79
		TURBT	98	99	98
		Normal	93.16	100	95.97
ResNet34	WLI	Above T1	84	84	84
		TA	93	70	80
		TURBT	97	98	97
		Normal	96.5	100	98.1
	SAVE	Above T1	90	88	89
		TA	84	80	82
		TURBT	99	99	99
		Normal	98.3	100	99.1
YOLOv5	WLI	Above T1	93.8	93.8	93.8
		TA	90	90	90
		TURBT	100	100	100
		Normal	100	100	100
	SAVE	Above T1	81.2	81.2	81.2
		TA	85	85	85
		TURBT	100	100	100
		Normal	100	100	100

## Data Availability

The data presented in this study are available on request from the corresponding author. The data are not publicly available due to strict privacy and ethical restrictions mandated by the Institutional Review Board of Kaohsiung Armed Forces General Hospital (KAFGHIRB 114-021).

## Supplementary Materials

The following supporting information can be downloaded at https://www.mdpi.com/article/10.3390/cancers18132147/s1, Figure S1: Comparative analysis of precision for five classification algorithms (VGG16, GoogLeNet, InceptionResNetV2, ResNet34, and YOLOv5) across WLI and SAVE imaging modalities. Figure S2: Comparative analysis of recall for five classification algorithms (VGG16, GoogLeNet, InceptionResNetV2, ResNet34, and YOLOv5) across WLI and SAVE imaging modalities. Figure S3: Comparative analysis of F1-score for five classification algorithms (VGG16, GoogLeNet, InceptionResNetV2, ResNet34, and YOLOv5) across WLI and SAVE imaging modalities. Figure S4: Confusion matrices for ResNet34 using WLI and SAVE imaging. Figure S5: Classification reports for ResNet34 using WLI and SAVE imaging. Figure S6: Confusion matrices for VGG16 using WLI and SAVE imaging. Figure S7: Classification reports for VGG16 using WLI and SAVE imaging. Figure S8: Confusion matrices for GoogLeNet using WLI and SAVE imaging. Figure S9: Classification reports for GoogLeNet using WLI and SAVE imaging. Figure S10: Confusion matrices for InceptionResNetV2 using WLI and SAVE imaging. Figure S11: Classification reports for InceptionResNetV2 using WLI and SAVE imaging. Figure S12: Classification reports for YOLOv5 using WLI and SAVE imaging. Figure S13: SSIM comparison between simulated NBI (SAVE) and WLI images for VCE and Olympus systems. Figure S14: Entropy comparison between simulated NBI and WLI images of Olympus endoscopy and VCE camera. Figure S15: PSNR comparison of twenty randomly selected images from Olympus and VCE systems. Figure S16: Visual comparison of imaging techniques: (a) WLI, (b) NBI, and (c) SAVE. Table S1: SSIM values for twenty randomly selected images from VCE and Olympus endoscopes. Table S2: Entropy comparison between WLI and NBI images for Olympus and VCE endoscopes. Table S3: PSNR values for twenty randomly selected images from Olympus and VCE endoscopes.
