# Supplementary material for: Early Classification of Bladder Cancer Using Spectrum-Aided Visual Enhancer (SAVE) and Deep Learning Models: A Non-Invasive Technology for Faster Detection
Source: Cancers (Basel). 2026 Jul 3;18(13):2147. doi: 10.3390/cancers18132147 (PMC13359820; doi:10.3390/cancers18132147)
Supplement: Supplementary file 1 [file cancers-18-02147-s001.zip › cancers-4331738-supplementary.pdf]

Supplementary Materials

Early Classification of Bladder Cancer Using Spectrum-Aided Visual Enhancer (SAVE) and Deep Learning Models: A Non-Invasive Technology for Faster Detection

Min-Hsin Yang [1,2], Yaswanth Nagisetti [3], Arvind Mukundan [4,5], Riya Karmakar [6], Chun-Feng Chang [7], Syna Syna [3,8], Ying-Jui Ni [7,*] and Hsiang-Chen Wang [3,9,*]

[1] Institute of Medicine, Chung Shan Medical University, 402 No. 110, Section 1, Jianguo North Road, Taichung 40201, Taiwan; barbarian0607@icloud.com

[2] Department of Urology, Chung Shan Medical University Hospital, 402 No. 110, Section 1, Jianguo North Road, Taichung 40201, Taiwan

[3] Department of Mechanical Engineering, National Chung Cheng University, 168, University Rd., Minxiong Township, Chia Yi 62102, Taiwan; yaswanthnagisatti@gmail.com (Y.N.); synab6498@gmail.com (S.S.)

[4] Department of Biomedical Engineering, Chennai Institute of Technology, Chennai 600069, Tamil Nadu, India; arvindmukund96@gmail.com

[5] Department of Computer Science Engineering, School of Engineering and Technology, Sanjivani University, Kopargaon 423603, Maharashtra, India

[6] Department of Integrated B.Tech, School of Engineering and Technology, Sanjivani University, Kopargaon 423603, Maharashtra, India; karmakarriya345@gmail.com

[7] Department of Surgery, Urological Surgery Division, Kaohsiung Armed Forces General Hospital, 2, Zhongzheng 1st Rd., Lingya District, Kaohsiung 80284, Taiwan; ccf701221@gmail.com

[8] Department of Computer Science and Engineering, Chitkara University, Chandigarh-Patiala National Highway NH-64 Village Jansla, Rajpura 140401, Punjab, India

[9] Department of Technology Development, Hitspectra Intelligent Technology Co., Ltd., Kaohsiung 80661, Taiwan

* Correspondence: niyingjui@gmail.com (Y.-J.N.); hcwang@ccu.edu.tw (H.-C.W.)

S1. Evaluation Indices

Evaluation metrics provide quantitative measures to objectively assess and compare the performance of machine learning models. In this study, we utilized the following key indices:

- **Accuracy:** Measures the overall proportion of correctly classified instances out of the total dataset. (Equation (S1))

$$\text{Accuracy} = \frac{TP+TN}{TP+TN+FP+FN} \qquad \text{(S1)}$$

- **Precision:** Quantifies the exactness of the model, defined as the proportion of true positive predictions among all positive predictions. (Equation (S2))
- **Recall (Sensitivity):** Measures the completeness of the model, defined as the proportion of actual positive cases that were correctly identified. (Equation (S2))
-

$$\text{Precision} = \frac{tp}{tp+fp} \qquad \text{Recall} = \frac{tp}{tp+fn} \qquad \text{(S2)}$$

- **F1-Score:** The harmonic mean of precision and recall. It provides a more balanced evaluation metric than accuracy, particularly when dealing with class imbalance and uneven false positive/false negative distributions. (Equation (S3))

$$F = 2 \cdot \frac{\text{precision} \cdot \text{recall}}{\text{precision} + \text{recall}} \quad \text{(S3)}$$

- **Mean Average Precision (mAP):** For object detection (e.g., YOLOv5), average precision (AP) integrates the Precision-Recall curve. mAP is the mean of AP across all classes. Specifically, mAP50 evaluates detection performance at an Intersection over Union (IoU) threshold of 0.50, while mAP50-95 averages the performance across multiple IoU thresholds (0.50 to 0.95), reflecting the model's robustness in localizing challenging objects. (Equation (S4))

$$\text{mAP} = \frac{1}{N}\sum_{i=1}^{N} \text{AP}_i \quad \text{(S4)}$$

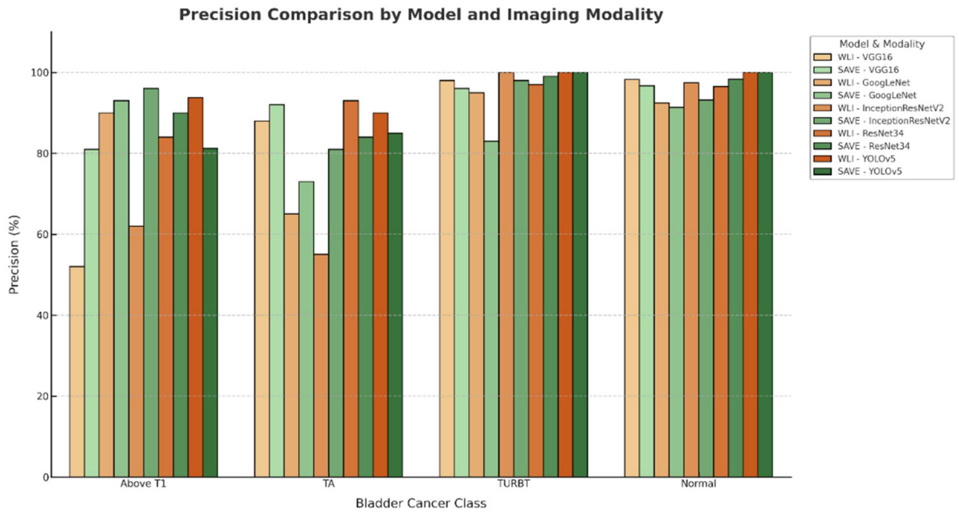

Figure S1. Comparative analysis of precision for five classification algorithms (VGG16, GoogLeNet, InceptionResNetV2, ResNet34, and YOLOv5) across WLI and SAVE imaging modalities.

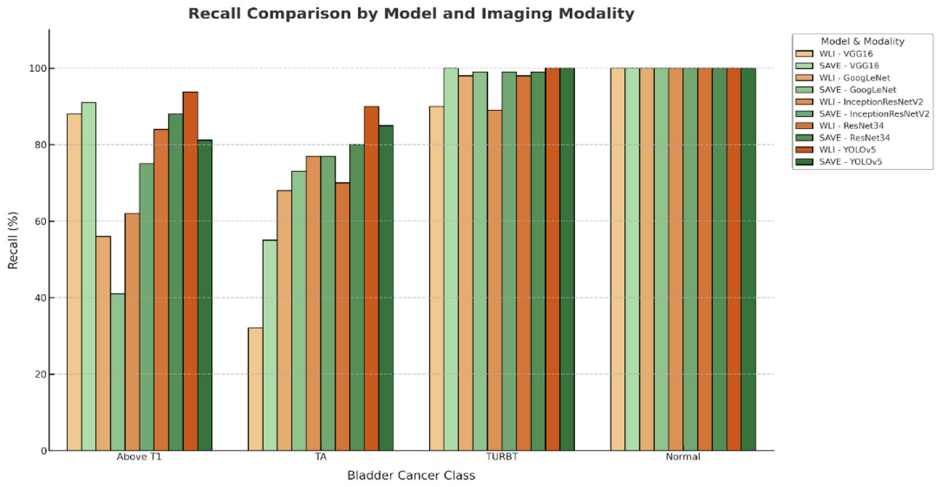

Figure S2. Comparative analysis of recall for five classification algorithms (VGG16, GoogLeNet, InceptionResNetV2, ResNet34, and YOLOv5) across WLI and SAVE imaging modalities.

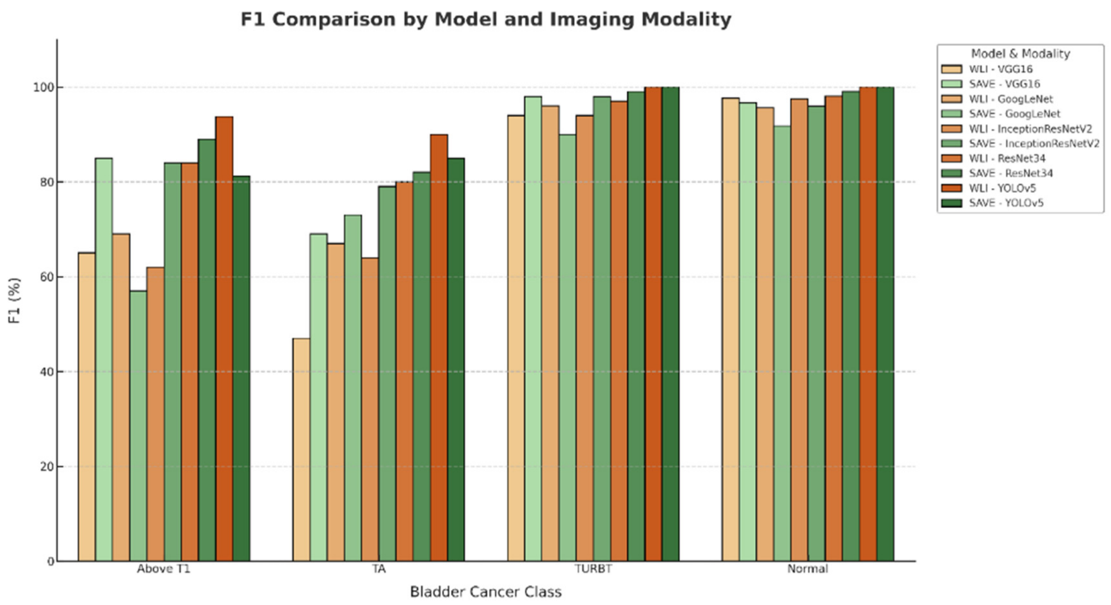

Figure S3. Comparative analysis of F1-score for five classification algorithms (VGG16, GoogLeNet, InceptionResNetV2, ResNet34, and YOLOv5) across WLI and SAVE imaging modalities.

S2. Spectrum-Aided Vision Enhancer (SAVE) Algorithm Details

The SAVE algorithm computationally transforms standard sRGB White Light Imaging (WLI) data into the XYZ color space using a systematic discrete conversion sequence.
First, the 24-color patch image and reflectance spectrum data from the sRGB color gamut are non-linearly transformed into the CIE 1931 XYZ color space using the following formulas:

$$\begin{bmatrix} X \\ Y \\ Z \end{bmatrix} = [M_A][T]\begin{bmatrix} f(R_{sRGB}) \\ f(G_{sRGB}) \\ f(B_{sRGB}) \end{bmatrix} \times 100\,, 0 \le \begin{matrix} R_{sRGB} \\ G_{sRGB} \\ B_{sRGB} \end{matrix} \le 1 \tag{S5}$$

$$[T] = \begin{bmatrix} 0.4104 & 0.3576 & 0.1805 \\ 0.2126 & 0.7152 & 0.0722 \\ 0.0193 & 0.1192 & 0.9505 \end{bmatrix} \tag{S6}$$

$$f(n) = \begin{cases} (\frac{n+0.055}{1.055})^{2.4}, n > 0.04045 \\ \left(\frac{n}{12.92}\right), otherwise \end{cases} \tag{S7}$$

$$[M_A] = \begin{bmatrix} X_{SW}/X_{CW} & 0 & 0 \\ 0 & Y_{SW}/Y_{CW} & 0 \\ 0 & 0 & Z_{SW}/Z_{CW} \end{bmatrix} \tag{S8}$$

$$Z = k\int_{400nm}^{700nm} S(\lambda)R(\lambda)\bar{z}(\lambda)d\lambda \tag{S9}$$

$$k = 100/\int_{400nm}^{700nm} S(\lambda)\bar{y}(\lambda)d\lambda \tag{S10}$$

Next, to account for hardware characteristics, the nonlinear response of the camera is adjusted using a third-order polynomial modification, denoted as $V_{Non-linear}$ (Equation (S11)). Simultaneously, a

constant value is assigned to represent the sensor's dark current contribution, established as V_{Dark} (Equation (S12)):

$$V_{Non-linear} = [X^3\ Y^3\ Z^3\ X^2\ Y^2\ Y^2\ X\ Y\ Z\ 1]^T \tag{S11}$$

$$V_{Dark} = [a] \tag{S12}$$

The final variable matrix V is obtained by standardizing the product of the color component (V_{Color}) and the non-linear component, followed by the addition of the dark current correction. To prevent over-correction, this standardization is strictly limited to the third order:

$$V_{Color} = [XYZ\ XY\ XZ\ YZ\ X\ Y\ Z]^T \tag{S13}$$

$$V = \begin{bmatrix} X^3\ Y^3\ Z^3 \\ X^2Y\ X^2Z\ Y^2Z \\ XY^2\ XZ^2\ YZ^2 \\ XYZ\ X^2\ Y^2\ Y^2 \\ XY\ XZ\ YZ\ X\ Y\ Z\ a \end{bmatrix}^T \tag{S14}$$

Finally, prior to computing the color difference using the CIE DE2000 standard, both the corrected XYZ values ($XYZ_{Correct}$) and the spectrum XYZ values ($XYZ_{Spectrum}$) must be converted from the XYZ color space into the CIELAB (L*ab**) color space. The conversion sequence is as follows:

$$\begin{aligned} L^* &= 116 f\left(\frac{Y}{Y_n}\right) - 16 \\ a^* &= 500\left[f\left(\frac{X}{X_n}\right) - f\left(\frac{Y}{Y_n}\right)\right] \\ b^* &= 200\left[f\left(\frac{Y}{Y_n}\right) - f\left(\frac{Z}{Z_n}\right)\right] \end{aligned} \tag{S15}$$

$$f(n) = \begin{cases} n^{\frac{1}{3}}, & n > 0.008856 \\ 7.787n + 0.137931, & otherwise \end{cases} \tag{S16}$$

S3. Results

S3.1 Results Visualization of RESNET34-WLI Model and RESNET34-SAVE Model

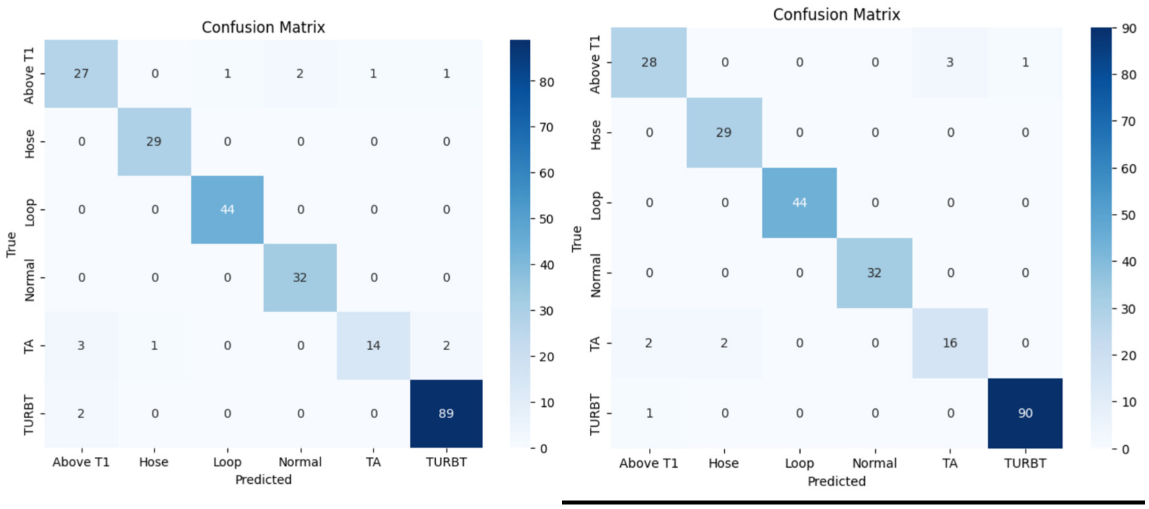

Figure S4. Confusion matrices for ResNet34 using WLI and SAVE imaging.

	precision	recall	f1-score	support
Above T1	0.84	0.84	0.84	32
Hose	0.97	1.00	0.98	29
Loop	0.98	1.00	0.99	44
Normal	0.94	1.00	0.97	32
TA	0.93	0.70	0.80	20
TURBT	0.97	0.98	0.97	91
accuracy			0.95	248
macro avg	0.94	0.92	0.93	248
weighted avg	0.95	0.95	0.95	248

	precision	recall	f1-score	support
Above T1	0.90	0.88	0.89	32
Hose	0.94	1.00	0.97	29
Loop	1.00	1.00	1.00	44
Normal	1.00	1.00	1.00	32
TA	0.84	0.80	0.82	20
TURBT	0.99	0.99	0.99	91
accuracy			0.96	248
macro avg	0.94	0.94	0.94	248
weighted avg	0.96	0.96	0.96	248

Figure S5. Classification reports for ResNet34 using WLI and SAVE imaging.

S3.2 Results Visualization of VGG16-WLI Model and of VGG16-SAVE Model

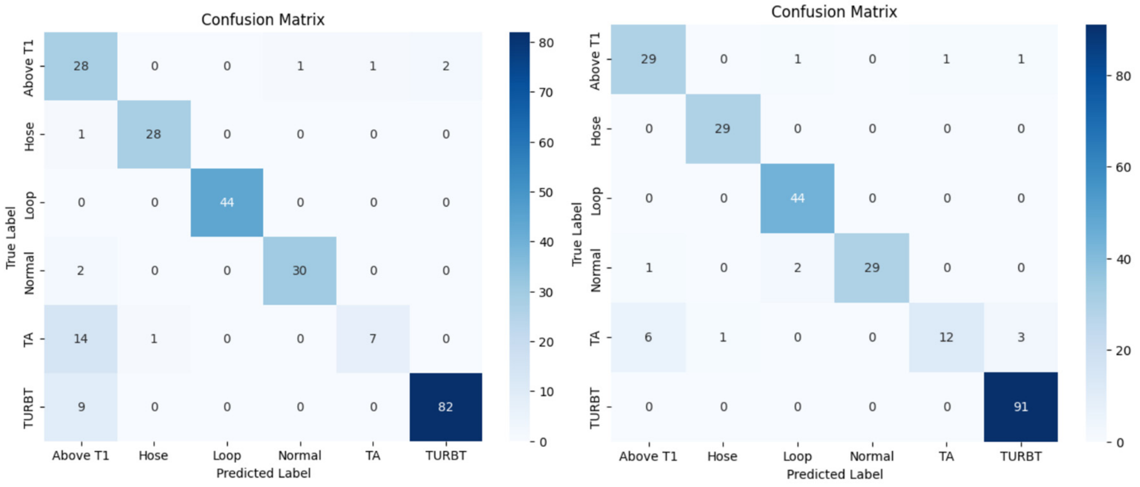

Figure S6. Confusion matrices for VGG16 using WLI and SAVE imaging.

	precision	recall	f1-score	support
Above T1	0.52	0.88	0.65	32
Hose	0.97	0.97	0.97	29
Loop	1.00	1.00	1.00	44
Normal	0.97	0.94	0.95	32
TA	0.88	0.32	0.47	22
TURBT	0.98	0.90	0.94	91
accuracy			0.88	250
macro avg	0.88	0.83	0.83	250
weighted avg	0.91	0.88	0.88	250

Classification Report

	precision	recall	f1-score	support
Above T1	0.81	0.91	0.85	32
Hose	0.97	1.00	0.98	29
Loop	0.94	1.00	0.97	44
Normal	1.00	0.91	0.95	32
TA	0.92	0.55	0.69	22
TURBT	0.96	1.00	0.98	91
accuracy			0.94	250
macro avg	0.93	0.89	0.90	250
weighted avg	0.94	0.94	0.93	250

Figure S7. Classification reports for VGG16 using WLI and SAVE imaging.

S3.3 Results Visualization of GoogLeNet-WLI Model and GoogLeNet-SAVE Model

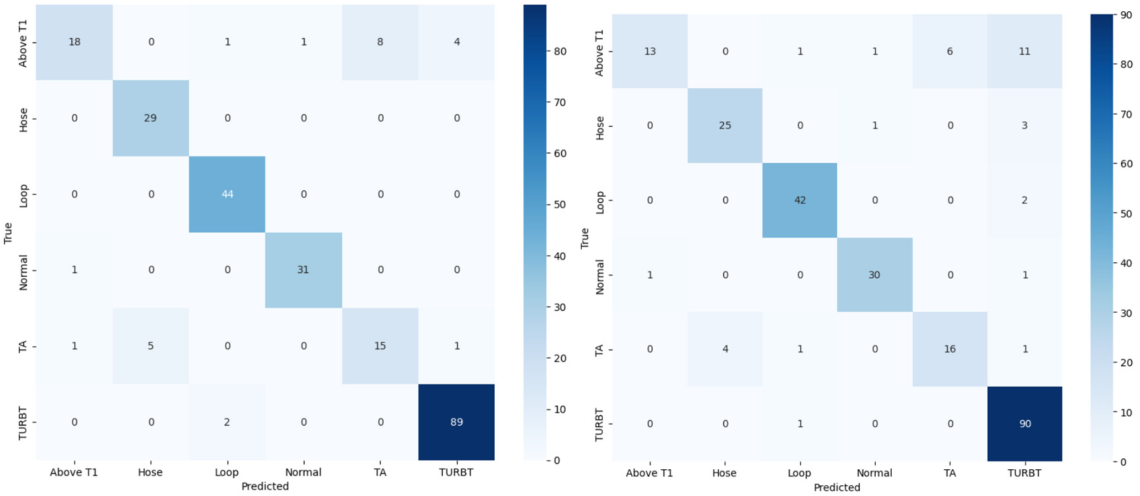

Figure S8. Confusion matrices for GoogLeNet using WLI and SAVE imaging.

	precision	recall	f1-score	support
Above T1	0.90	0.56	0.69	32
Hose	0.85	1.00	0.92	29
Loop	0.94	1.00	0.97	44
Normal	0.97	0.97	0.97	32
TA	0.65	0.68	0.67	22
TURBT	0.95	0.98	0.96	91
accuracy			0.90	250
macro avg	0.88	0.87	0.86	250
weighted avg	0.90	0.90	0.90	250

	precision	recall	f1-score	support
Above T1	0.93	0.41	0.57	32
Hose	0.86	0.86	0.86	29
Loop	0.93	0.95	0.94	44
Normal	0.94	0.94	0.94	32
TA	0.73	0.73	0.73	22
TURBT	0.83	0.99	0.90	91
accuracy			0.86	250
macro avg	0.87	0.81	0.82	250
weighted avg	0.87	0.86	0.85	250

Figure S9. Classification reports for GoogLeNet using WLI and SAVE imaging.

S3.4 Results Visualization of InceptionResNetV2-WLI Model vs InceptionResNetV2-SAVE Model

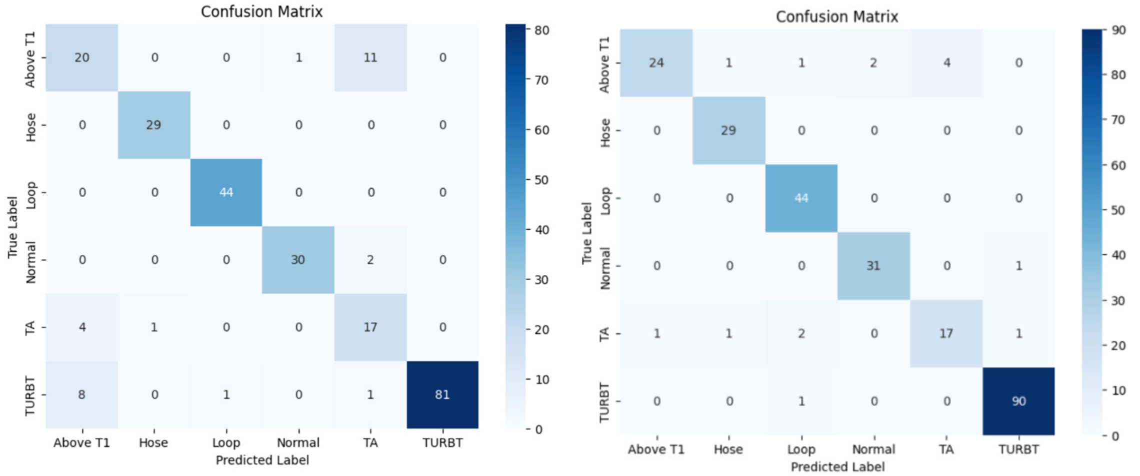

Figure S10. Confusion matrices for InceptionResNetV2 using WLI and SAVE imaging.

	precision	recall	f1-score	support
Above T1	0.62	0.62	0.62	32
Hose	0.97	1.00	0.98	29
Loop	0.98	1.00	0.99	44
Normal	0.97	0.94	0.95	32
TA	0.55	0.77	0.64	22
TURBT	1.00	0.89	0.94	91
accuracy			0.88	250
macro avg	0.85	0.87	0.86	250
weighted avg	0.90	0.88	0.89	250

	precision	recall	f1-score	support
Above T1	0.96	0.75	0.84	32
Hose	0.94	1.00	0.97	29
Loop	0.92	1.00	0.96	44
Normal	0.94	0.97	0.95	32
TA	0.81	0.77	0.79	22
TURBT	0.98	0.99	0.98	91
accuracy			0.94	250
macro avg	0.92	0.91	0.92	250
weighted avg	0.94	0.94	0.94	250

Figure S11. Classification reports for InceptionResNetV2 using WLI and SAVE imaging.

S3.5 Results Visualization of YOLOv5-WLI Model and YOLOv5-SAVE Model

Class	Images	top1_acc	top5_acc
all	248	0.984	1
Above T1	32	0.938	1
Hose	29	1	1
Loop	44	1	1
Normal	32	1	1
TA	20	0.9	1
TURBT	91	1	1

pre-process, 2.2ms inference, 0.1ms post-process per image at shape
ı to runs/val-cls/exp

Class	Images	top1_acc	top5_acc
all	248	0.964	0.992
Above T1	32	0.812	0.969
Hose	29	1	1
Loop	44	1	1
Normal	32	1	1
TA	20	0.85	0.95
TURBT	91	1	1

ıs pre-process, 2.2ms inference, 0.1ms post-process per image at:
ved to runs/val-cls/exp

Figure S12. Classification reports for YOLOv5 using WLI and SAVE imaging.

S4. Evaluation of SAVE

The valuation of the SAVE algorithm is comprehensively assessed through its performance metrics, specifically the Structural Similarity Index Metric (SSIM), pixel signal to noise ratio (PSNR) and entropy, which provide quantitative insights into the algorithm's effectiveness in image reproduction. At first SSIM between the simulated and the real WLI of the Olympus endoscope can be compared with the SAVE image from the HSI conversion algorithm developed in this study. SSIM can be defined as the amount of similarities between the simulated and the real image. The values range between 0% to 100% where 100% means the images are completely the same and 0% means the images are completely different. It is measured for the images that are reconstructed from a base image. Therefore, this parameter fits our criteria of comparison. Similarly, the WLI image of the VCE can also be compared with the SAVE images obtained from the HSI conversion algorithm. Figure S10 shows the

SSIM for both the Olympus images and the VCE images. It can be seen that the Olympus images have a better SSIM rate with an average of 94.27% while the VCE has a comparatively lesser. Still, the average SSIM for VCE was found to be around 90%. This is because for NBI the CIEDE 2000 color calibration was possible for the traditional endoscope with the real NBI. After all, there was a reference real NBI image available. But for VCE no such reference is available. So, the same calibration that was done for the NBI images of the Olympus was done for VCE. Even though without any reference the algorithm achieved a SSIM of 90%. It can also be seen that the top three highest achieved SSIM values was from the VCE of 96%. From this, we can infer that the results of the study are accurate. In this study 50 randomly chosen VCE images were used for calculating the SSIM. However, by increasing the number of images the SSIM can be significantly improved. Table S1 shows the SSIM comparison of twenty randomly chosen images in VCE and Olympus endoscope.

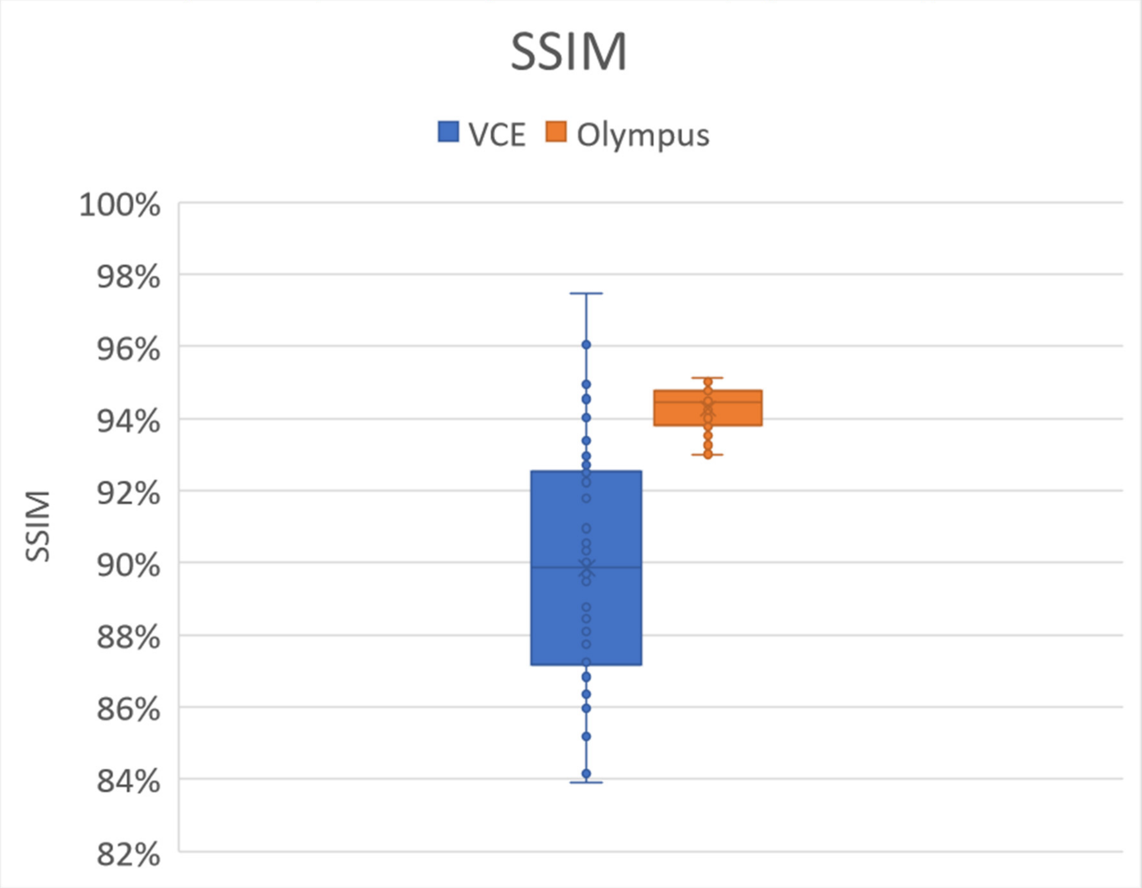

Figure S13. SSIM comparison between simulated NBI (SAVE) and WLI images for VCE and Olympus systems.

Table S1. SSIM values for twenty randomly selected images from VCE and Olympus endoscopes.

Index	SSIM in VCE	SSIM in Olympus
1	0.925984794	0.94883467
2	0.944862636	0.948624384
3	0.928211385	0.94554478
4	0.966524653	0.937769205
5	0.92933774	0.941602499
6	0.958101429	0.945524249
7	0.939473779	0.935284698
8	0.936779292	0.933442268

9	0.872748906	0.938402164
10	0.873266671	0.936314054
11	0.891602213	0.934205211
12	0.944929655	0.949261138
13	0.805573811	0.941545835
14	0.90568673	0.946374234
15	0.83298981	0.945601764
16	0.912076221	0.932277471
17	0.936323837	0.930091206
18	0.792020729	0.939289678
19	0.936141641	0.932559509
20	0.903283862	0.935927389
Avg.	90.680%	93.992%

The second criterion that was used to evaluate the algorithm developed in this study was entropy. The entropy was also calculated similarly to the SSIM. The difference in entropy between the WLI images obtained from the Olympus endoscope is compared with the SAVE images simulated from the HSI conversion algorithm. In image processing, entropy might be used to classify textures, a certain texture might have a certain entropy as certain patterns repeat themselves in approximately certain ways. In the context of the paper low entropy means low disorder, low variance within the component. Therefore, lower the entropy better reproduction of the image is obtained. The difference in entropy between the WLI images obtained from VCE is compared with the SAVE images from the HSI-NBI conversion algorithm. Figure S11 shows the entropy difference in Olympus endoscope and VCE. As it can be seen from Table S2 the entropy difference in both the VCE and the Olympus endoscope have similar values. The average entropy difference in VCE was 1.17% while the average difference in the Olympus endoscope was 0.37%. However, in VCE the majority difference was found to be caused by only one image (image number 11). If we remove that image the entropy difference value is just 0.03% which is better than the Olympus endoscope. Table S2 shows the entropy comparison of the WLI and SAVE images in Olympus and VCE endoscope of twenty random images.

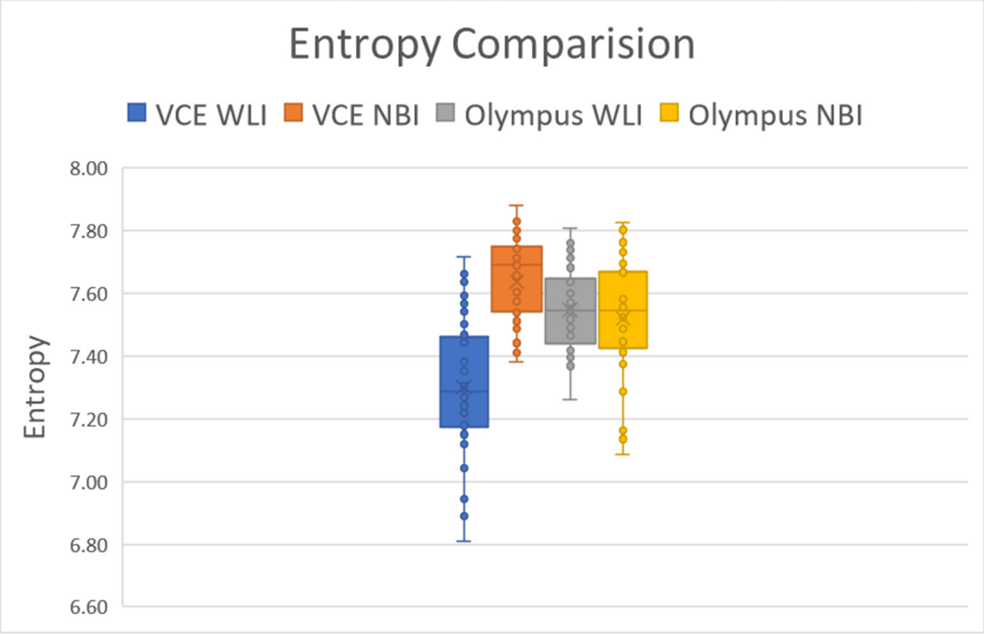

Figure S14. Entropy comparison between simulated NBI and WLI images of Olympus endoscopy and VCE camera.

Table S2. Entropy comparison between WLI and NBI images for Olympus and VCE endoscopes.

Index	VCE		Endoscope		Difference in VCE	Difference in Olympus
	WLI	NBI	WLI	NBI		
1	7.04162	7.213345	7.60896	7.576404	0.024387	-0.004298
2	7.14502	7.220075	7.56380	7.494327	0.010504	-0.009271
3	7.37728	7.396152	7.65457	7.522734	0.002557	-0.017526
4	7.35948	7.278474	7.41264	7.283070	-0.011008	-0.017791
5	7.30720	7.363200	7.63545	7.680663	0.007663	0.005886
6	7.25352	7.350173	7.57114	7.546219	0.013325	-0.003167
7	6.88228	7.182308	7.53630	7.557263	0.043593	0.002773
8	7.25791	7.345877	7.71332	7.804702	0.012119	0.011708
9	6.95063	6.713150	7.56143	7.669351	-0.034167	0.014072
10	7.04094	7.134570	7.57816	7.676892	0.013297	0.012860
11	7.21094	6.575100	7.52565	7.372113	-0.088178	-0.020827
12	7.21771	7.219500	7.43596	7.422078	0.000247	-0.001871
13	7.58770	7.112978	7.57403	7.671311	-0.062566	0.012680
14	7.17281	7.184375	7.55415	7.667614	0.001612	0.014797
15	7.23312	7.210437	7.63470	7.693241	-0.003137	0.007608
16	7.14741	7.384973	7.71922	7.729203	0.033237	0.001291
17	7.28176	7.259479	7.75930	7.761126	-0.003061	0.000234
18	7.18062	6.801497	7.74727	7.801845	-0.052799	0.006994
19	7.26419	7.290983	7.74532	7.825175	0.003688	0.010205
20	7.59704	7.555495	7.736022	7.807877	-0.005469	0.009203
Avg	7.2254647	7.189607	7.61332	7.628160	-0.47%	0.19%

PSNR is typically utilized in the context of image compression algorithms as a parameter to evaluate the quality of the reproduced image. In a comparison of quality that is comparable to that of the SSIM, the PSNR values of twenty randomly selected WLI images and their SAVE equivalents are measured. The plot of the PSNR for each of the twenty images is displayed in Figure S12. The PSNR of the VCE images came in at an average of 28.0216 db, while the PSNR of the Olympus images was 27.8819 db. Table S3 shows the comparison of PSNR of the twenty randomly chosen images in Olympus and VCE

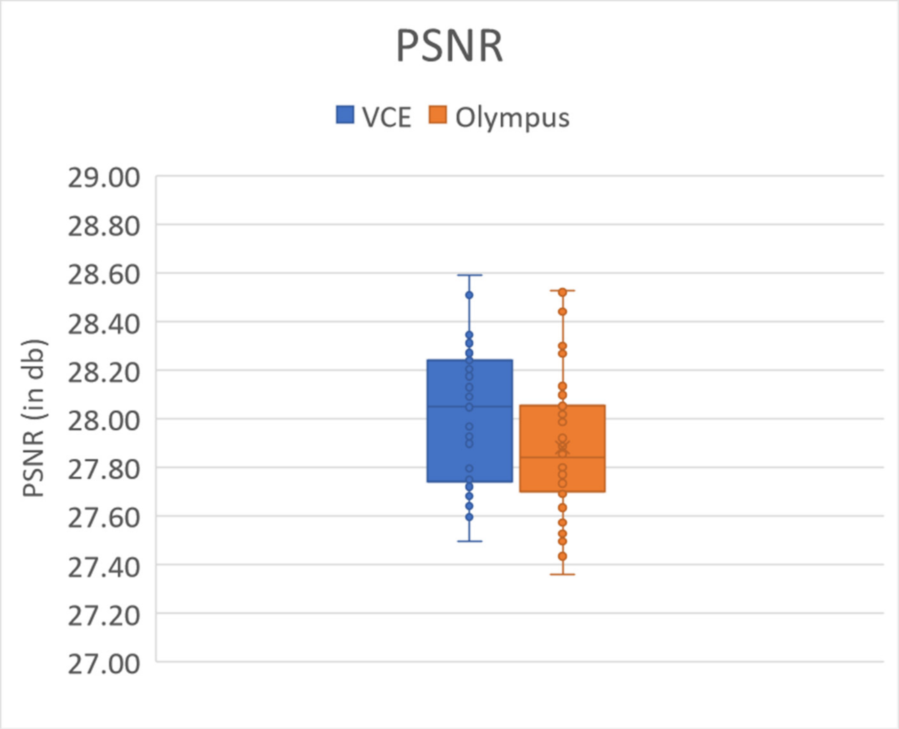

Figure S15. PSNR comparison of twenty randomly selected images from Olympus and VCE systems.

Table S3. PSNR values for twenty randomly selected images from Olympus and VCE endoscopes.

Index	PSNR of Olympus images	PSNR of VCE images
1	27.5737493	28.51448883
2	27.4460567	27.72258326
3	27.46222796	27.66114567
4	27.36380762	27.49849716
5	27.82132106	27.67697859
6	27.49987858	28.1142441
7	27.78251246	27.90179575
8	27.71866018	27.6486571
9	27.78163772	27.65547302
10	27.63666623	28.0393251
11	27.58337722	28.20507339
12	27.70590967	27.99117518
13	27.53574671	28.26982928
14	27.68741185	27.9901207
15	27.74701762	28.26732897
16	27.81360865	27.7055983
17	27.87955844	27.90082759
18	27.71300104	28.50043856
19	27.73324857	27.68852332
20	28.02414942	27.68115621
Avg.	27.67547735	27.931663

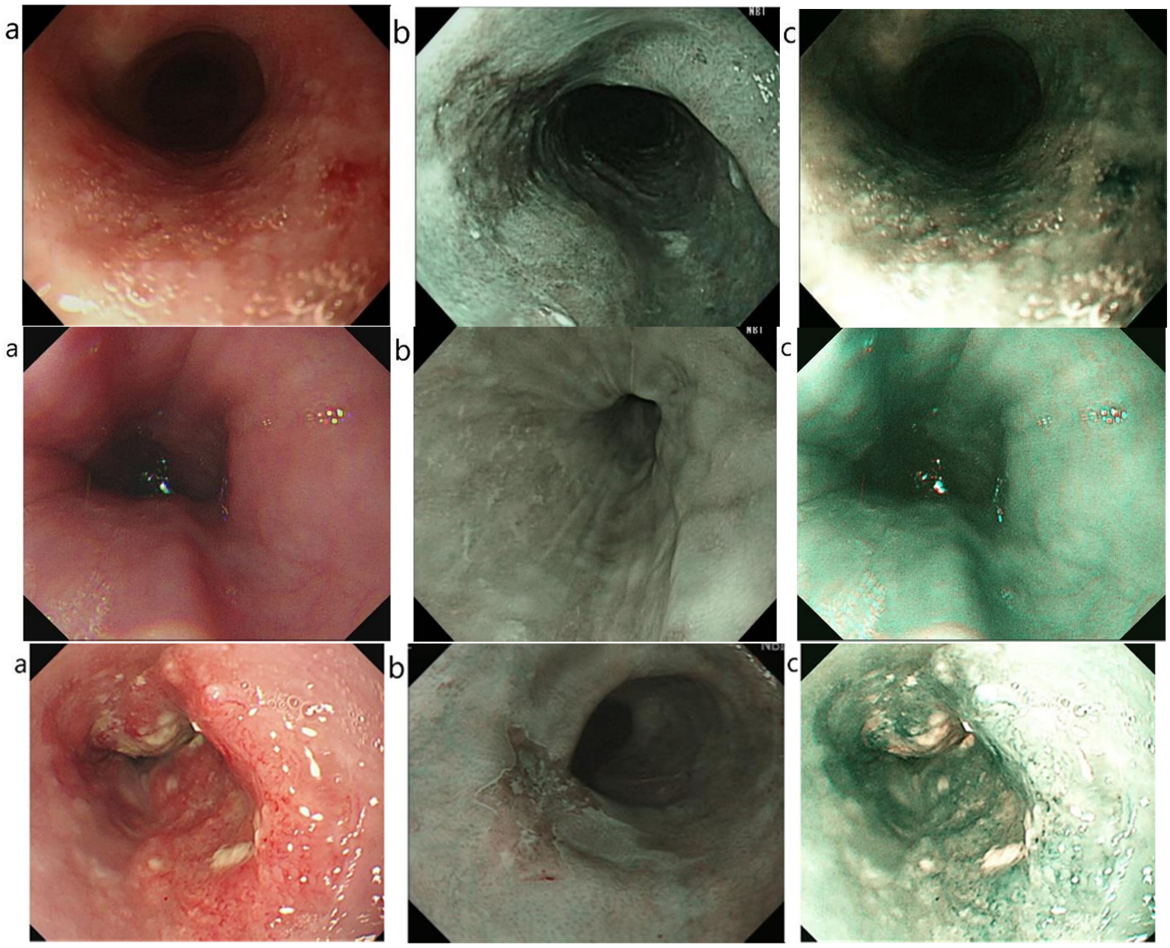

Figure S16. Visual comparison of imaging techniques: (a) WLI, (b) NBI, and (c) SAVE.
